# Calcium Channel Blockers: Key Medicine to Drive Global Hypertension Control

**DOI:** 10.5334/gh.1443

**Published:** 2025-07-14

**Authors:** Andrew E. Moran, Swagata Kumar Sahoo, Bolanle Banigbe, Sohel Reza Choudhury, Prabhdeep Kaur, Renu Garg

**Affiliations:** 1 Resolve to Save Lives, New York, USA; 2 Columbia University Irving Medical Center, New York, USA; 3 National Heart Foundation Hospital and Research Institute, Dhaka, Bangladesh; 4 Isaac Centre for Public Health, Division of Medical Sciences, Indian Institute of Science, Bangalore, India

**Keywords:** hypertension, antihypertensive medicine, calcium channel blocker, global health, low- and middle-income country, team-based care, patient-centered care, medicine access

## Background

Globally, less than half of the patients with hypertension (HTN) receive pharmacological treatment, and one in every five has their blood pressure controlled (1). About 80% of people with HTN reside in low- or middle-income countries (LMICs), where control rates are less than 10% (1).

The most important cause of low HTN control rates in LMICs is limited access to essential antihypertensive medicines. The World Health Organization (WHO) recommends that most HTN patients are treated with antihypertensive drugs from one or more of three main first-line classes: long-acting dihydropyridine calcium channel blockers (CCBs), renin–angiotensin–aldosterone system antagonists (angiotensin-converting enzyme inhibitors [ACEis] or angiotensin 2 receptor blockers [ARBs]), and thiazide and thiazide-like diuretics (TZDs). Because of constrained government spending on HTN medicines and limited or no insurance coverage of medicine costs, many HTN patients in LMICs pay out of pocket for their antihypertensive medicines and high out-of-pocket cost is a major barrier. Another access barrier is the lack of laboratory testing recommended to detect adverse effects of ACEis, ARBs, and TZDs. ACEi and ARB implementation are also complicated because these are known teratogens and contraindicated in female HTN patients who might become pregnant. There is reluctance in some countries to prescribe TZDs because of the rare risk of dehydration, hypokalemia leading to cardiac arrhythmias, and possibly increased risk of skin cancer. By contrast, CCBs are similarly efficacious and avoid barriers associated with the other recommended classes.

Since 2017, the multi-partner Global Hearts Initiative has worked to overcome barriers to HTN control in LMICs, including medicine access. It supports the implementation of the WHO HEARTS technical package for HTN control, which includes (1) linear, stepwise drug and dose-specific treatment protocols and (2) a reliable supply of protocol drugs. By 2024, over 34 million HTN patients were enrolled in HEARTS-based treatment across 38 LMICs. Despite variable country contexts, most HEARTS HTN treatment protocols recommend initiating CCB at protocol Step 1. We set out to describe current clinical and implementation factors favoring the primacy of CCBs in HTN treatment programs in LMICs.

### CCBs: Efficacy and Safety

#### CCB efficacy

CCBs inhibit the entry of calcium ions into vascular smooth muscle cell membrane channels, leading to vasodilation, which lowers systemic arterial resistance and blood pressure. Landmark monotherapy trials found comparable efficacies for all four WHO-recommended drug classes. CCBs are slightly better at preventing stroke (2). They are frequently included in multi-class antihypertensive drug single-pill combinations such as dual therapy (combined with one other major class) or triple therapy (combined with two other classes) (3,4).

Pragmatic implementation trials in LMIC primary care settings demonstrated that monotherapy protocols starting with CCB are effective in controlling blood pressure and are feasible to implement. The quasi-experimental Bangladesh HEARTS trial in 14 primary care clinics showed that an initial CCB regimen, as part of the WHO-HEARTS technical package, led to >12% higher HTN control compared with usual care (5). The CREOLE trial enrolled 728 hypertensive patients from 6 sub-Saharan African countries in a three-arm trial of dual combination antihypertensive treatments (6). CREOLE found that CCB-based dual therapy (CCB+ACEi or CCB+TZD) led to >3 mmHg blood pressure lowering compared with ACEi+TZD. The similarly designed TOPSPIN trial found that dual combinations including CCB had similar efficacy and safety compared with an ACEi+TZD combination in 1981 HTN patients recruited at sites across India (7). The TRIUMPH and VERONICA trials, conducted in HTN patients living in Sri Lanka and Nigeria, respectively, demonstrated similar safety and superior blood pressure-lowering efficacy with an initial low-dose CCB+ARB+TZD combination protocol compared with initial monotherapy protocols (8,9).

#### CCB safety

A main advantage of CCBs is that they are metabolically neutral and, unlike other major antihypertensive classes, cause no adverse effects on serum sodium, potassium, or glomerular filtration rate. Consequently, no pre- or post-treatment laboratory monitoring is required after starting a CCB. The main side effect of CCBs is dose-dependent benign pedal edema owing to increased fluid permeability of the capillary circulation, which can be reversed by stopping or lowering the dose of the CCB. In a first systematic review of randomized, controlled trials from high-income countries, peripheral edema incidence attributable to CCB was 7.5% (10.7% CCB vs. 3.2% placebo, P < 0.0001) (10). In a second review, peripheral edema incidence was 10.4% (16.6% CCB vs. 6.2% placebo, P < 0.0001) (11). Peripheral edema increased at higher doses (e.g., 16.1% with high-dose CCB and 5.7% with low-dose CCB) (10). In the first review, the rate of drug withdrawal due to edema was lower than the rate of any edema: 2.1% in participants taking CCB compared with 0.5% in those taking placebo (10).

Recently, the rates of pedal edema from CCBs have been reported for HTN populations in LMICs. CREOLE, in which most participants receiving CCB took a maximal dose (10 mg amlodipine), found that pedal edema leading to drug withdrawal was 3–4% higher with dual therapy including CCB compared with non-CCB treatment (ACEi+TZD) (9). In the TOPSPIN trial, leg swelling leading to drug withdrawal was only 1% higher in the arms including CCB treatment compared with the non-CCB combination (12). In a single-arm, pre-post study of blood pressure and metabolic effects of initiating a CCB+ARB combination (amlodipine 5 mg + telmisartan 40 mg) in 864 HTN patients living in Dhaka, Bangladesh, the incidence of pedal edema after starting CCB+ARB was only 0.6% (13). Overall, the rate of pedal edema leading to CCB discontinuation is low, dose-dependent, and reversible.

### Implementation of CCB-Based HTN Treatment in LMICs: The Global Hearts Initiative Experience

The Global Hearts Initiative recommends that countries develop simple HTN treatment protocols using a consensus process engaging HTN patients, local clinical and pharmacy experts, and government stakeholders (14).

Due to efficacy and safety and no need for laboratory monitoring, availability, affordability, and patient-centeredness, CCBs were selected as a first-step medicine on HEARTS monotherapy and dual therapy protocols developed by almost all country consensus conferences (Table 1) (15). Of 50 Global Hearts HTN treatment protocols recorded before mid-2023, 44/50 (88%) included a CCB as part of the first protocol step, and 13/50 (26%) recommended maximal dose CCB (e.g., amlodipine 10 mg) as the second protocol step (15). Initial CCB monotherapy is a reasonable choice for settings without laboratory testing capacity and constrained medicine procurement budgets. For countries favoring initial dual-drug therapy, CCBs are included in almost all protocols: of 23 Global Hearts protocols that recommended initial dual-drug combination, 17 included CCB in the combination.

**Table 1:** Summary of CCB role in HEARTS HTN control programs in low- and middle-income countries (LMICs).


FACTOR	HEARTS EXPERIENCE

**Effectiveness**	HEARTS protocol including initial CCB more effective than usual care without preferred protocol medicines (5) Comparable HTN control rate

**Role in simple treatment protocol**	88% of HEARTS HTN treatment protocols include CCB at Step 1 (either monotherapy or part of dual therapy) (15) 26% of HEARTS HTN treatment protocols recommend maximal dose CCB monotherapy at the second protocol step (15)

**Safety**	Many LMIC PHC facilities lack laboratory testing; CCBs cause no metabolic adverse effects and do not require laboratory testing The main CCB side effect is pedal edema, which is dose-dependent and reverses with CCB discontinuation

**Affordability**	Limited affordability of antihypertensive medicines in many LMICs (16) As monotherapies, CCBs are among the most available and affordable antihypertensive medicines in LMICs (16) However, prices of single-pill combinations including CCBs are often not affordable in all LMICs (17)

**Availability**	CCB monotherapies are available for purchase for public and private sector payers in LMICs (16) Because CCBs can be used to manage HTN in pregnancy, these agents are often more reliably available in PHCs that provide peripartum care Single-pill combinations including CCBs are not consistently available in LMICs

**Patient-centered care**	CCBs are stable across a range of temperatures, and due to no need for laboratory monitoring they can be dispensed by non-physician health workers at the community clinic level, as in the Bangladesh Community-Based HEARTS study

**Research gaps**	Head-to-head comparisons of HEARTS protocol variations to assess comparative effectiveness and safety of maximal dose CCB monotherapy regimens Implementation studies are needed to evaluate implementation barriers and facilitators of CCB-containing dual and triple combination therapies


CCB-based protocols have been part of successful HEARTS package implementation. A pre-post “sentinel sites” pragmatic HEARTS HTN control program evaluation in almost 160,000 HTN patients representing 23 districts in the Punjab and Maharashtra states of India found that among patients retained in the program, outcomes with the use of CCB for the first two treatment steps were comparable to protocols adding a second medication class at Step 2 (18). Specifically, the Punjab state protocol with amlodipine 5 mg at Step 1 followed by amlodipine 10 mg at Step 2 controlled blood pressure <140/90 mmHg in 75% of patients in Step 2, an outcome comparable to the 81% controlled outcome with the Maharashtra state protocol that also started with amlodipine 5 mg but added an ARB (telmisartan 40 mg) at Step 2.

CCBs can facilitate patient-centered HTN care. CCB-first HTN treatment protocols (e.g., amlodipine 5 mg at Step 1, followed by amlodipine 10 mg at Step 2 if not controlled at Step 1) mean that for initial management, only one medicine must be stocked, and no laboratory testing services are needed. Nurses or other non-physician health workers staffing community-based clinics (the most local-level facilities) can be trained to manage the first two steps of CCB-centered protocols with minimal supervision. The ongoing Community-Based HEARTS study is testing the efficacy of HTN diagnosis and initial management by community health workers using a CCB-first protocol in four rural community clinics in Bangladesh (19). Patients are started on amlodipine 5 mg and escalated to 10 mg if they remain uncontrolled. Patients uncontrolled on amlodipine 10 mg are referred to a higher-level facility for additional medicines and management. Implementation and effectiveness outcomes from the pilot phase of this study will be reported in 2025.

LMICs urgently need more affordable antihypertensive medicines in general, and CCBs are currently among the most available and affordable options. CCBs are almost always included on LMIC national essential medicine lists (16,17). LMIC public sector CCB prices are sometimes slightly higher than, but often close to, the estimated cost-based generic price standard; once-daily CCBs are often priced below one US penny per day or less than US$4 per year (17). In terms of affordability for HTN patients, 30 days of CCB in the public sector costs 3–5-day wages of low-wage workers, which compares favorably with other antihypertensive medicine classes (16). Despite the advantages of CCBs, past national surveys found only about one-third or less of LMIC public sector health-care facility pharmacies stocked long-acting dihydropyridine CCBs (amlodipine or nifedipine) (16). Single-pill combinations that include a CCB component are not yet widely available in LMICs, especially in public sector pharmacies; market-shaping work is needed to reduce the price of single-pill combinations that include a CCB component (17).

## Discussion

In summary, CCBs are among the three preferred antihypertensive medicine classes recommended by the WHO. For many LMIC settings, CCBs have the added advantages over other classes in LMIC settings based on their equivalent effectiveness and safety, and lack of need for laboratory monitoring, availability, affordability, and role in patient-centered HTN care. The advantages of CCBs naturally led to their primary role in HEARTS simple HTN treatment protocols around the world. As the most prescribed HTN medicine in Global Hearts programs, CCBs have the potential to transform the market for antihypertensive medicines in LMICs. The ubiquity of CCBs as part of first-step therapy across countries and regions could lead to an “economy of scale,” ushering in low medicine prices needed to accelerate the scale-up of HTN control programs in LMICs. CCBs, as monotherapy or part of combination therapy, can drive the growth of HTN control programs in LMICs to a national and global scale. To realize this scale, the urgent need is to prioritize CCB availability and affordability for all people living with HTN.
